# Handling Permutation in Sequence Comparison: Genome-Wide Enhancer Prediction in Vertebrates by a Novel Non-Linear Alignment Scoring Principle

**DOI:** 10.1371/journal.pone.0141487

**Published:** 2015-10-27

**Authors:** Dirk Dolle, Juan L. Mateo, Michael P. Eichenlaub, Rebecca Sinn, Robert Reinhardt, Burkhard Höckendorf, Daigo Inoue, Lazaro Centanin, Laurence Ettwiller, Joachim Wittbrodt

**Affiliations:** 1 Centre for Organismal Studies (COS) Heidelberg, Ruprecht-Karls-University Heidelberg, Heidelberg, Germany; 2 Hartmut Hoffmann-Berling International Graduate School HBIGS, University of Heidelberg, Heidelberg, Germany; Pohang University of Science and Technology (POSTECH), REPUBLIC OF KOREA

## Abstract

Enhancers have been described to evolve by permutation without changing function. This has posed the problem of how to predict enhancer elements that are hidden from alignment-based approaches due to the loss of co-linearity. Alignment-free algorithms have been proposed as one possible solution. However, this approach is hampered by several problems inherent to its underlying working principle. Here we present a new approach, which combines the power of alignment and alignment-free techniques into one algorithm. It allows the prediction of enhancers based on the query and target sequence only, no matter whether the regulatory logic is co-linear or reshuffled. To test our novel approach, we employ it for the prediction of enhancers across the evolutionary distance of ~450Myr between human and medaka. We demonstrate its efficacy by subsequent *in vivo* validation resulting in 82% (9/11) of the predicted medaka regions showing reporter activity. These include five candidates with partially co-linear and four with reshuffled motif patterns. Orthology in flanking genes and conservation of the detected co-linear motifs indicates that those candidates are likely functionally equivalent enhancers. In sum, our results demonstrate that the proposed principle successfully predicts mutated as well as permuted enhancer regions at an encouragingly high rate.

## Introduction

To date, two main, more or less diametrically opposed structural models of enhancers exist. The first model describes so-called enhanceosomes, regions of densely clustered transcription factor binding sites (TFBSs) that require a tightly coordinated series of binding events of partially interacting transcription factors (TFs) [1]. Due to this rigid structure, such cis-regulatory elements (CREs) tolerate only limited sequence changes without affecting their functionality. Hence, this class of enhancers is likely to be detected by alignment-based sequence comparison methods and further by methods assessing evolutionary constraint. Indeed, methods looking for conserved non-coding sequences (CNS), often defined as regions of >70% sequence conservation across >100nt [2–5], have been applied for the task of enhancer prediction and in many cases yielded elements capable of driving reporter gene expression in various spatiotemporal patterns in a variety of organisms. Nobrega et al. for example, scanned the human gene desert around the *DACH1* gene for regions conserved between human, mouse, frog, and zebrafish and found several elements recapitulating parts of the known *DACH1* expression pattern that are likely to be functional [4]. Pennacchio et al. expanded this approach on a genome-wide scale following two different strategies [2]. They searched for elements which either are conserved (70% identity, >100nt) across >450Myr of independent evolution between human and fugu, or conserved between human, mouse, and rat. Due to the much smaller phylogenetic distance between the species in the latter approach, the authors employed a more stringent criterion: 100% sequence identity over at least 200nt in all three species (“ultraconserved elements” (UCEs)) [6]. Both approaches identified CREs at rates between 29% and 61%, providing further support for the applicability of alignment-based strategies for enhancer prediction. Although the significance of ultraconservation has been questioned since then [7,8], there is no doubt that sequence conservation and evolutionary constraint are very useful tools for the genome-wide prediction of regulatory elements. To date, thousands or CREs have been identified this way, most of them located next to transcription factor or developmental genes (“trans-dev genes” [5]).

However, almost 30 years ago [9] the first hints were provided that evolution and diversification of species might occur by changes on the regulatory rather than on the coding level, and today an increasing body of evidence supports this hypothesis [9–15]. Hence, another class of regulatory elements, with a higher level of sequence divergence, must play an important role in the evolution of expression of these genes. Possible candidates are enhancers following the second main structural model of CREs: the “billboard model”. These regions consist of flexible arrangements of TFBS that, upon binding of the corresponding TFs, start to aggregate and form the final active enhancer complex [16–18]. Contrary to the rather rigid structure of enhanceosomes, such arrangements are more easily modified without leading to a complete loss of function–a property that would allow regulators of crucial developmental genes to evolve. Indeed, there is strong evidence that billboard enhancers are able to keep most of their activity even in case of large structural rearrangements and strong binding site turnover [19,20], allowing a gradual adaptation to a new/extended role without critically affecting the organism as a whole. However, over time this inherently flexible nature is likely to render them invisible for classical alignment-based approaches and hence prevents their detection. This property can therefore be described as the intrinsic technical challenge of studying enhancer evolution.

One early approach to tackle this problem was to focus on the functional elements of CREs: the TFBS. Regulatory regions that retain their function even after structural rearrangements sufficient to hide them from sequence alignments still need to provide interaction sites for the TFs involved in activation of the enhancer. The same holds true for regions with modified activity. Although the profile of involved TFs might change in this scenario, it is unlikely that all sites for factors used previously are lost. Hence, looking for clusters of TFBS in a given genome has the potential to reveal otherwise covert elements and several studies have used this approach for enhancer prediction [21–24]. Some of the more sophisticated approaches among them use a combination of TFBS prediction and alignment algorithms to solve this problem. For instance, He et al. developed a method that uses a probabilistic model of TFBS evolution to align two regions in different species based on the predicted positions of corresponding binding sites and applied this technique for the classification of known regulatory regions in *Drosophila melanogaster* [25]. In a similar way, Taher et al. aligned putative regulatory regions to a known enhancer based on predicted TFBS–profiles [26]. This allowed them to detect enhancers in likely orthologous regions across the evolutionary distance between human and zebrafish. While successful, both methods rely on prior knowledge of TFBSs for enhancer prediction and hence cannot be applied in contexts where this information is not available. Sosinsky et al. were able to overcome this necessity by developing a method that infers putative TFBS from multiple sequence alignments of closely related species [27]. Applied on a set of known regulatory regions in *D*. *melanogaster*, they successfully predicted the putative corresponding enhancers in the vicinity of orthologous genes in multiple fly species. Unfortunately, this method depends on the availability of identifiable orthologous regions in multiple closely related species and therefore cannot be applied for genome-wide enhancer prediction, especially if the target is a single distantly related species.

Another attempt to handle the flexible nature of enhancers employs word profiles. This class of algorithms is usually referred to as “alignment-free”, although TFBS clustering methods in principle could also be summarized under that term. For simplicity, we will restrict the use of this term to methods using the working mechanism described in the following section. The common principle of these techniques is the base-wise dissection of two sequences into words of a defined size k (also called “k-mers”), generating a word profile for each. This profile is then used for sequence comparison. One of the first applications of this methodology on a biological question was the search for similar candidates for a set of given genes in all available bacterial GenBank sequences [28]. Later approaches successfully used the comparison of word profiles for the reconstruction of phylogenetic trees based on protein sequences [29]. For a review of the variety of alignment-free techniques and their use for sequence comparison see [30]. The main difference between individual alignment-free methods is the metric used to compare the generated profiles. While the aforementioned methods used weighted word counts or the angle between two given word vectors for comparison, subsequent methods focused on expected word frequencies for similarity assessment [31–33]. Each of these methods has since then been applied to enhancer prediction in insects and vertebrates and successfully identified known or novel regulatory regions [34,35]. However, in most of these studies [31,32,35] an initial training set of enhancers of similar regulatory activity was necessary to extract the likely key features needed for enhancer prediction. Furthermore, in only one study [35] a true genome-wide scan for regulatory elements was performed, in insects in this case.

Realizing that alignment-free-based enhancer prediction so far has mainly been performed in flies, we wondered what hinders their application on a genome-wide scale in the more complex vertebrate genomes. We further sought to understand why most methods so far depend on various types of additional information, especially when trying to predict enhancers across large evolutionary distances, for instance between human and fish. We therefore analysed the alignment-free principle for possible limitations in the concept itself in order to develop compensation strategies that do not require additional information.

Here we propose a new principle of sequence similarity assessment that combines the ability of alignment algorithms to extract the maximum signal contained in two sequences with the ability of alignment-free techniques to cope with permutation. This principle was implemented in an experimental algorithm and subsequently applied for enhancer prediction on a genome-wide scale in the teleost medaka (*Oryzias latipes*). Using known and previously validated human enhancers as template, we found and tested 11 medaka regions of which nine show clear enhancer activity. Interestingly, subsequent analysis of the structural organisation of the validated predictions revealed that co-linear motif configurations as well as permuted arrangements are almost equally likely to show enhancer activity.

## Results

### A composite principle for enhancer prediction

Alignment-free techniques have been suggested and applied for enhancer prediction due to their theoretical ability to deal with permutation [30]. In practice, however, this ability is hampered already by the initial generation of the word profile. Extracting k-mers of a fixed size in an overlapping fashion greatly simplifies TFBSs as unambiguous sequences of equal length independent of the TF binding to it. It further allows individual words to change independently although every position in a word is overlapped by a series of others neighbouring it (see S1 File and S1 Fig). As previously mentioned, many methods published so far make use of additional information besides the given enhancer sequence in order to compensate the resulting problems [25,26] [27,32]. Here, we present a different approach with the aim of using exclusively the sequence of a known enhancer and a target genome. For this, we adapted a seeding and match/mismatch extension step for profile generation that first looks for short, perfect matching k-mers between two given sequences. Applying a mismatch tolerant scoring scheme, similar to those used by many local aligners, these "seeds" are then extended. By doing so, we can handle variable positions within putative TFBSs that otherwise would have led to different k-mers. At the same time, it allows us to reduce the size of the final profile by focusing only on extended seeds above a set threshold score. As regions extracted in this way are likely to be of different size and variable match/mismatch rates like the TFBSs they represent, we will call them “motifs” instead of “words”.

This seed extension approach is supported by previous studies which found that spacer sequences between individual TFBSs in cis-regulatory modules (CRMs) evolve faster than the functional regions they separate, slowing down gradually the closer a specific position is to a functional site [36]. Hence, ancestrally related TFBSs are more likely to allow extension in both directions than random occurrences of the same sites. In another paper, Swanson et al. [18] have shown that whole clusters of TFBSs can rearrange in the same way as individual TFBSs without violating the strict spacing and orientation requirements between the individual sites. As a result, even full clusters might be detectable as a single motif after rearrangement given that the TFBSs involved are close enough to each other and an initial seed can be placed.

### Pattern detection

The higher evolutionary constraint within CRMs compared to the surrounding sequence has consequences also on the CRE level. Like CRMs, which consist of a combination of TFBSs, CREs can be composed of several CRMs, each of them responsible for a specific spatial and/or temporal aspect of the full regulatory activity [16]. As the sequence between those modules evolves at a higher rate than the modules themselves, it can become non-alignable by series of mutations and indels while the CRMs are kept more or less conserved. This leads to a situation in which a continuous CRE is separated into a series of CRM blocks, with the CRMs arranged like beads on a chain. However, while the mutated sequence within CRMs can be bridged by mismatches in the extension step, the larger distances between CRMs cannot. Even gapped aligners are unlikely to handle this situation, as it would require the simultaneous introduction of gaps in both sequences. Chaining/Netting algorithms on the other hand, e.g. those used by the multiple aligner MultiZ [37], can identify these regions as a series of conserved blocks while ignoring the sequence between them. However, these algorithms usually require multiple genomic sequences containing the same enhancer. Furthermore, the individual CRMs need to be rather strongly conserved in all those genomes to be detectable.

In the light of these results, we developed a technique adapted from a gap detection mechanism initially implemented in the CHAOS aligner [38]. In short, the modified principle works by first defining a search space around a selected match in a given alignment and subsequently scanning the search space for other matches that can be incorporated in a co-linear pattern (for details see “Methods”). While this allows enhancing the similarity score using co-linear arrangements, it does not interfere with the detection of sequence permutation as long as the profile generation step is performed first. In fact we allow two types of predictions, one in which the pattern detection is not considered (“PURE” score) and other in which this technique is included (“COMB” score).

**Fig 1:**
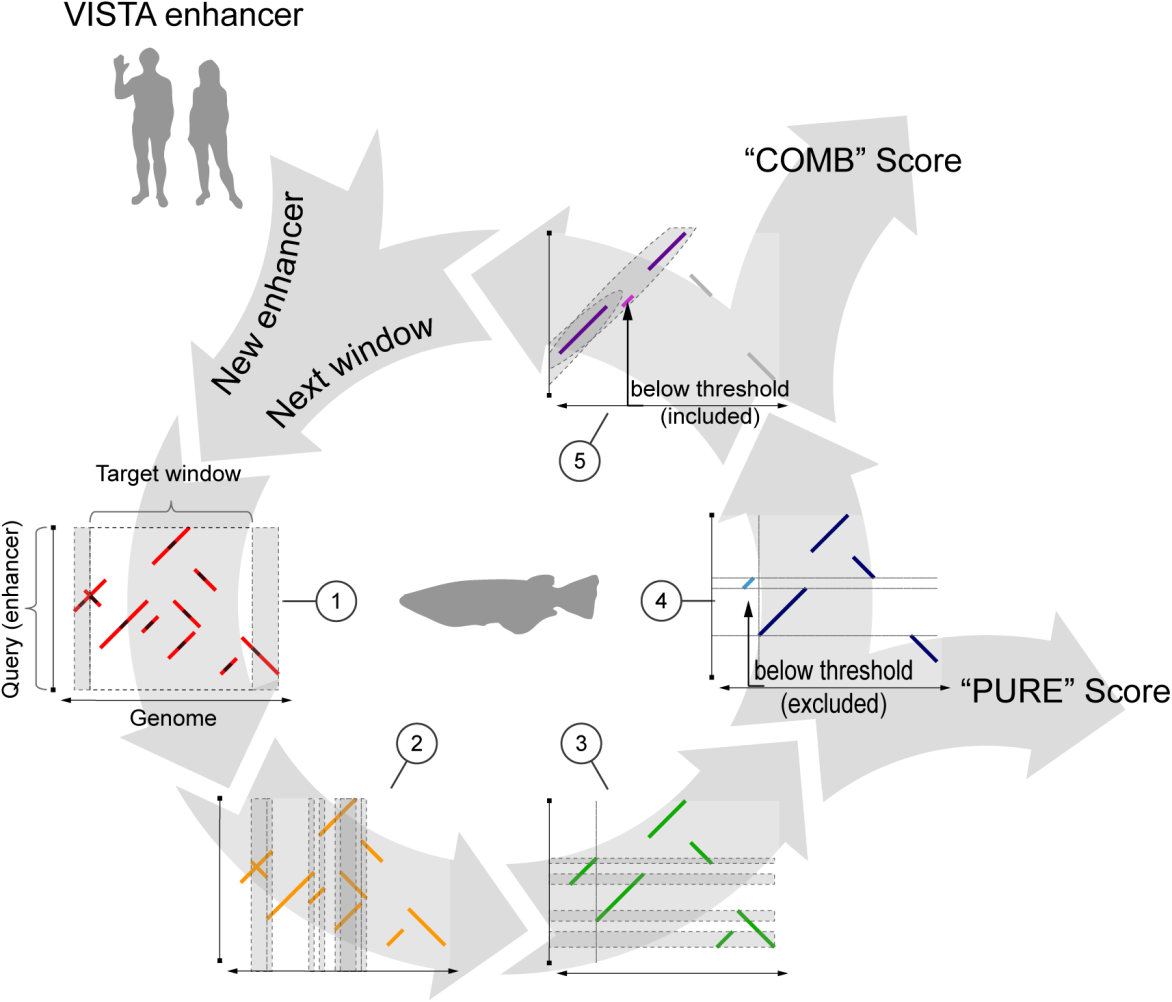
NASCAR workflow. (1) Seeds perfectly matching between query (i.e. enhancer) and target (e.g. genomic window) sequence (small black segments) are extended up- and downstream (red segments) using a match/mismatch scoring scheme to generate a raw motif profile. Motifs that overlap the predefined window boundaries are also taken into account and virtually extend the window (grey areas). (2) As a next step, overlapping regions of the extracted raw motifs in the target sequence are determined (grey areas) and the smaller motif truncated whenever it overlaps a larger one (2 to 3). Motifs smaller than the initial seed size after truncation are discarded in this step. (3) Same filtering procedure is repeated in the query sequence for the processed profile (3 to 4). (4) Motifs below the noise threshold (bright blue segment) are discarded and the basic similarity (“PURE”) score calculated from the fully filtered motif profile (dark blue). (5) In addition, a pattern detection method searches for co-linear arrangements in the profile (grey area). Panel shows the same motif composition as (4) but in a co-linear configuration. This time, the motif below the noise threshold (bright pink) is kept as it is contained in a pattern. The score of the full pattern (all pink motifs) is subsequently added to the previously calculated basic score, resulting in the “COMB” score. For a given enhancer, the whole process is repeated window by window until the last window in the target sequence is reached.

Finally, we combined seed extension and pattern detection into a single composite algorithm called NASCAR (“Non-linear Alignment SCoring AlgoRithm”), which can be used for genome-wide sequence similarity searches. Like most alignment-free algorithms, it looks for regions matching a given input sequence by sliding a window across the target genome. For each window defined in this way, a motif profile is generated and used to assess similarity to the input sequence (Fig 1).

NASCARs source code (written in PERL) is available upon request.

### Selection of a test set of known enhancers

To further analyse the extent to which the principle described here can be used for the task of enhancer prediction, we applied it to a dataset of known and validated human enhancers. These regions were extracted from the VISTA Enhancer Browser [39] (http://enhancer.lbl.gov/), perhaps the biggest collection of *in vivo* validated vertebrate enhancers similar to the RedFly database [40]. For this study, we selected all human enhancers that were reported to have enhancing activity at E11.5 in mouse and removed overlapping regulatory elements (see Methods). This resulted in 629 enhancers that were used for all subsequent analyses.

Most of these enhancers were initially predicted by deep sequence conservation, including sequences from amphibians and partially even fish, and hence likely to align in the majority of sequenced vertebrates. We therefore selected the teleost medaka (*Oryzias latipes*) as the target species in this study, for which a reliable enhancer assay exists [41,42]. Like all teleosts, the medaka genome shows clear signs of an additional whole genome duplication event (WGD) when compared to tetrapods, which happened shortly after the teleost-tetrapod split ~350Myr ago. The resulting redundancy on both the gene and the regulatory level is thought to have allowed formerly conserved enhancers to evolve, provided at least one functional copy is retained. A subsequent loss of one of the two instances, e.g. due to the chromosomal restructuring events that followed the duplication, would re-establish the selective pressure on the remaining enhancer and hence fix it in its current state. In some cases, this could have been the faster evolving copy while the more conserved instance was lost, leaving no trace of a regulatory element in the teleosts that is still conserved in tetrapods. Hence, trying to bridge the ~450Myr of independent evolution of human and medaka represents a challenging task when considering that enhancers might have undergone permutation events to an extent that could render them invisible to alignment algorithms. However, this type of CREs could be uncovered by NASCAR.

To enrich our study set for CREs that could have undergone the scenario described above, we first sorted out all enhancers that are still alignable across the evolutionary distance between human and medaka. For this, we applied two local alignment algorithms, LastZ [43] and BlastN [44], on the full dataset, resulting in alignments for 252 of the 629 (~40%) enhancers for LastZ and 303 (~48%) for BlastN. To test whether both algorithms identify similar subsets or actually predict independent parts of the input set we overlapped the prediction results and found overlapping alignment hits for 248 enhancers, meaning that BlastN is missing only four (~2%) of the LastZ hits while making significant predictions for additional 55 enhancers. We therefore decided to use the regions identified by BlastN as the aligning set for further comparisons.

To ensure that this set is indeed a suitable reference we analysed the gene environment of all contained alignment hits and found that for 279 of 303 (~92%) enhancers the hit is located near human-medaka orthologs in medaka (S1 Table). In 160 cases (~53%), these alignments are located in direct flanking position to a gene that is orthologous between human and medaka, and we denoted these predictions as “single flanked”. Further 71 predictions (~23%) are positioned between both orthologous flanking genes, labelled as “double flanked”. The remaining 48 hits are near the medaka ortholog to a gene in the human locus (for the computation of orthologous gene sets see “Methods” and S4 Fig). However, even the 24 predictions that seem to be in “deserted” locations (i.e. without any orthologous gene nearby) are partially next to paralogs, which were not included in the analysis. Only a few predictions among those 24 seem to be completely remote. This suggests that the proximity of a pair of highly similar non-coding sequences to genes orthologous between both species can be used as an indication for the orthology of the found sequence match.

### NASCAR identifies aligning enhancers

We then tested NASCAR on the aligning data set to assess its sensitivity on a collection of likely true positive regions. For this, we first compared the highest scoring NASCAR predicted regions against the full set of alignments found by BlastN and could recover hits for 275 of 303 enhancers (~91%), including weakly aligning (bit score between 50–80) and gap containing regions. This shows that NASCAR is able to recover regions of overall low sequence similarity (lowest scoring overlapping BlastN hit: 70nt (4% coverage) at 79.4% percentage identity, bit score 51.8). Besides the highest scoring prediction per enhancer, NASCAR outputs additional regions that could also contain CREs. However, in contrast to BlastN, there is so far no significance threshold that could be used to expand the candidate set beyond the highest ranked region. But as the BlastN results show (BlastN gives 480 regions for 303 independent enhancers when applying a bit score cut-off of > = 50), many human enhancers have more than just one likely corresponding candidate in medaka (e.g. due to the WGD or segmental duplications). We therefore decided to consider the 25 highest NASCAR predictions as putative enhancer candidates for this very first test of the implemented principles. Based on this cut-off, NASCAR identifies BlastN hits for 283 (~93%) human enhancers. Interestingly, the fraction of BlastN alignments reported by NASCAR is already noticeably higher than the one found by LastZ (91% vs 83%) when using only the highest scoring NASCAR hit.

### Novel predicted candidates show reporter activity

Next, we applied NASCAR on the non-aligning 52% (326/629) of the VISTA enhancer set and analysed the reported predicted regions to identify putative enhancer candidates. Because of the obvious lack of alignment hits for this set, we developed a different assessment scheme based on the location of the hits relative to their flanking genes

In the aligning set, ~92% of all BlastN predictions in medaka are near to genes that are orthologous between human and medaka. Furthermore, in ~76% (231 of 303) of these predictions the hit in medaka is flanked by at least one gene orthologous to a gene flanking the human enhancer. For predictions in the non-aligning set we hence used the stricter latter criterion to select putative enhancer candidates among the 25 highest scoring NASCAR predictions per enhancer. This resulted in 221 regions near orthologous genes, with 18 even in directly flanking position. However, as we used an arbitrary cut-off of 25 for initial selection we decided to remove candidates that theoretically could also have been found by alignment algorithms following this procedure albeit at score levels not considered significant. Hence, we first selected the 25 highest BlastN alignments per input enhancer irrespective of their bit score. Afterwards, we filtered the set of non-aligning enhancers by removing all those with a selected NASCAR prediction close to an orthologous medaka gene in case this predicted regions was within 5kb of a BlastN hit. Of the remaining enhancers, 56 have a NASCAR prediction near the ortholog of a human flanking gene, nine of them even in flanking position in medaka. These nine are the highest confidence prediction set and were subsequently used for further tests *in vivo*.

**Fig 2:**
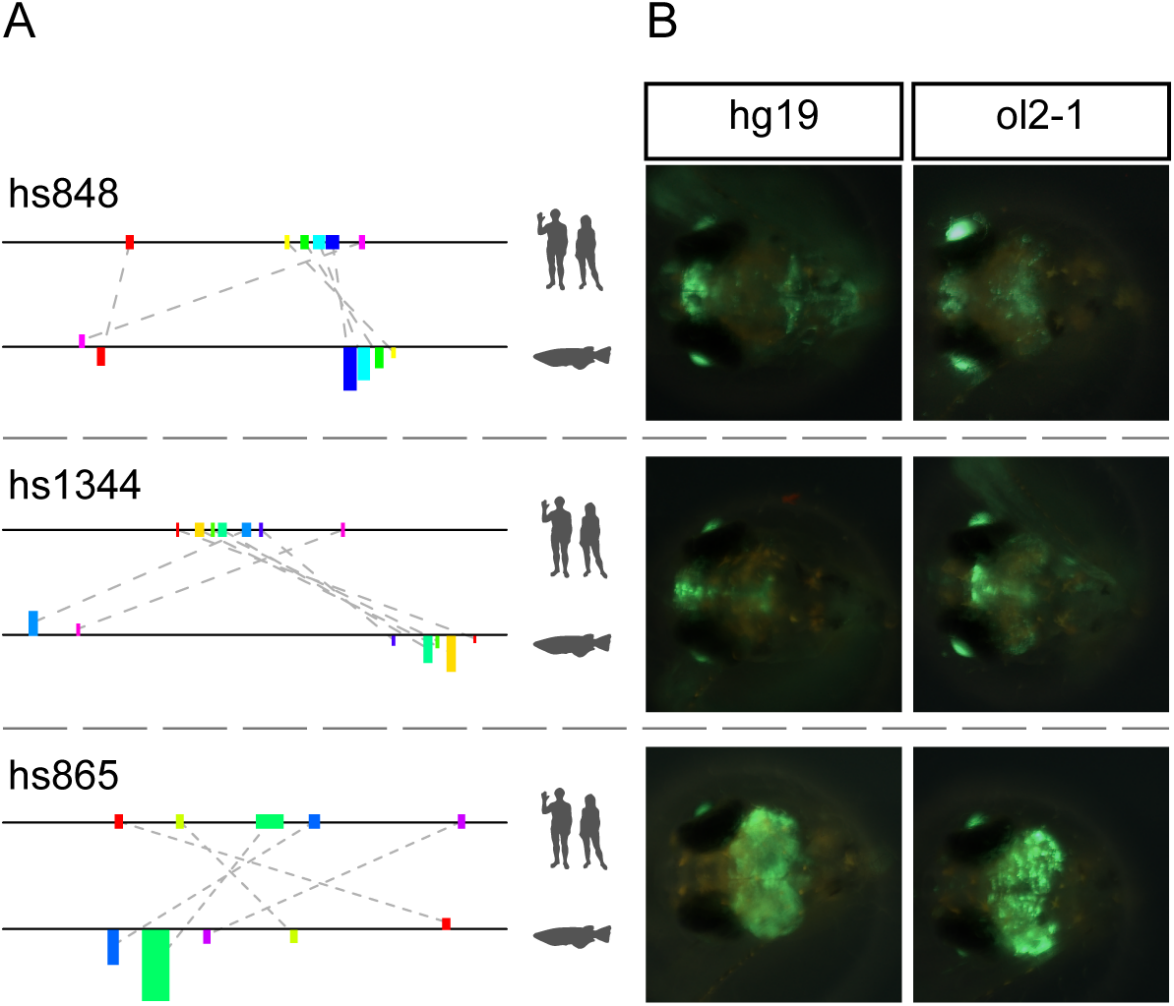
Motif composition and reporter gene activity of selected human VISTA enhancers. For all enhancers see S5 Fig. (A) Comparison of the known human enhancer sequence and the predicted enhancer in medaka. The coloured boxes represent the motifs identified by NASCAR to assess the similarity of each pair. Upper track always displays the motif positions in the human sequence (colour coded by position), lower track shows the configuration in the medaka region. All Motifs are draw in size relative to the used window size. Motif heights in the lower track represent the motif score, orientation (up/down) indicates the relative orientation (forward/reverse) compared to the query sequence. (B) Expression pattern of the human (hg19) or medaka (ol2-1) enhancers. Lens activity is part of the reporter construct and allows distinguishing between successful and negative injections. All pictures are taken at 10 days post injection (10dpi). In all cases both enhancers show strikingly similar pattern.

As a first step, the human region of each of the nine predicted enhancer pairs was validated using our reporter assay [41]. Of those, eight showed reporter gene activity in medaka (Table 1, Fig 2, S5A Fig), indicating that the trans environment in our model system is able to activate the regulatory potential encoded in the human sequences. Furthermore, the displayed activity pattern of most of the tested elements largely resembles the pattern found in mouse (S5A Fig), suggesting that not only the binding motifs for the factors involved but also the regulatory logic (i.e. the spatiotemporal control of gene expression driven by this set of factors) remained largely unchanged despite the large evolutionary distance between the species. Hence, we subsequently tested the medaka regions predicted for those eight active human enhancers and could confirm activity for six (75%) of them (S5A Fig). This rate of observed reporter activity is already very encouraging by itself, especially when compared to what is achieved by randomly selected regions (10%) [45], regions picked by clustering of Transfac motifs (54%) [2], or prediction using chromatin feature-based computational genomic segmentation (59%) [42] It is also higher than the 44% achieved by prediction based on deep sequence conservation [2]. More interesting, however, is the observation that the activity pattern driven by corresponding pairs partially overlaps in domains in the fore- and midbrain. This strongly suggests that the approach implemented in NASCAR detected components of the regulatory logic contained in the human enhancer regions and used them for prediction of the validated candidates.

**Table 1:** Selected NASCAR candidates.

VISTA ID	Human region	Size	Medaka region	Flank status	Rank
**hs1344** *	*hg19*:*3*:*193660817–193662478*	1662 nt	*ol2*:*4*:*13569031–13570692*	DF	2
**hs865** *	*hg19*:*6*:*50685244–50686237*	994 nt	*ol2*:*24*:*19553675–19554668*	DF	4
**hs1535**	*hg19*:*2*:*60498057–60502013*	3957 nt	*ol2*:*15*:*7226825–7230781*	DF	7
**hs848** *	*hg19*:*16*:*51491799–51493025*	1227 nt	*ol2*:*3*:*29251639–29252865*	SF	1
**hs1049** *	*hg19*:*5*:*92314781–92316083*	1303 nt	*ol2*:*9*:*15278121–15279423*	SF	1
**hs882** *	*hg19*:*13*:*71533037–71534195*	1159 nt	*ol2*:*21*:*9408987–9410145*	SF	1
**hs1831**	*hg19*:*7*:*95236622–95240458*	3837 nt	*ol2*:*11*:*9757778–9761614*	SF	3
**hs590**	*hg19*:*18*:*34719386–34720720*	1335 nt	*ol2*:*5*:*16698603–16699937*	SF	9
**hs394**	*hg19*:*2*:*59746377–59746992*	616 nt	*ol2*:*15*:*7017013–7017628*	DF	23

The first 8 entries correspond to the candidates that showed expression in medaka.

* = partial motif co-linearity, DF = “double flanked”, SF = “single flanked”

### Predicted motifs show higher conservation than random motifs in the same region

**Fig 3:**
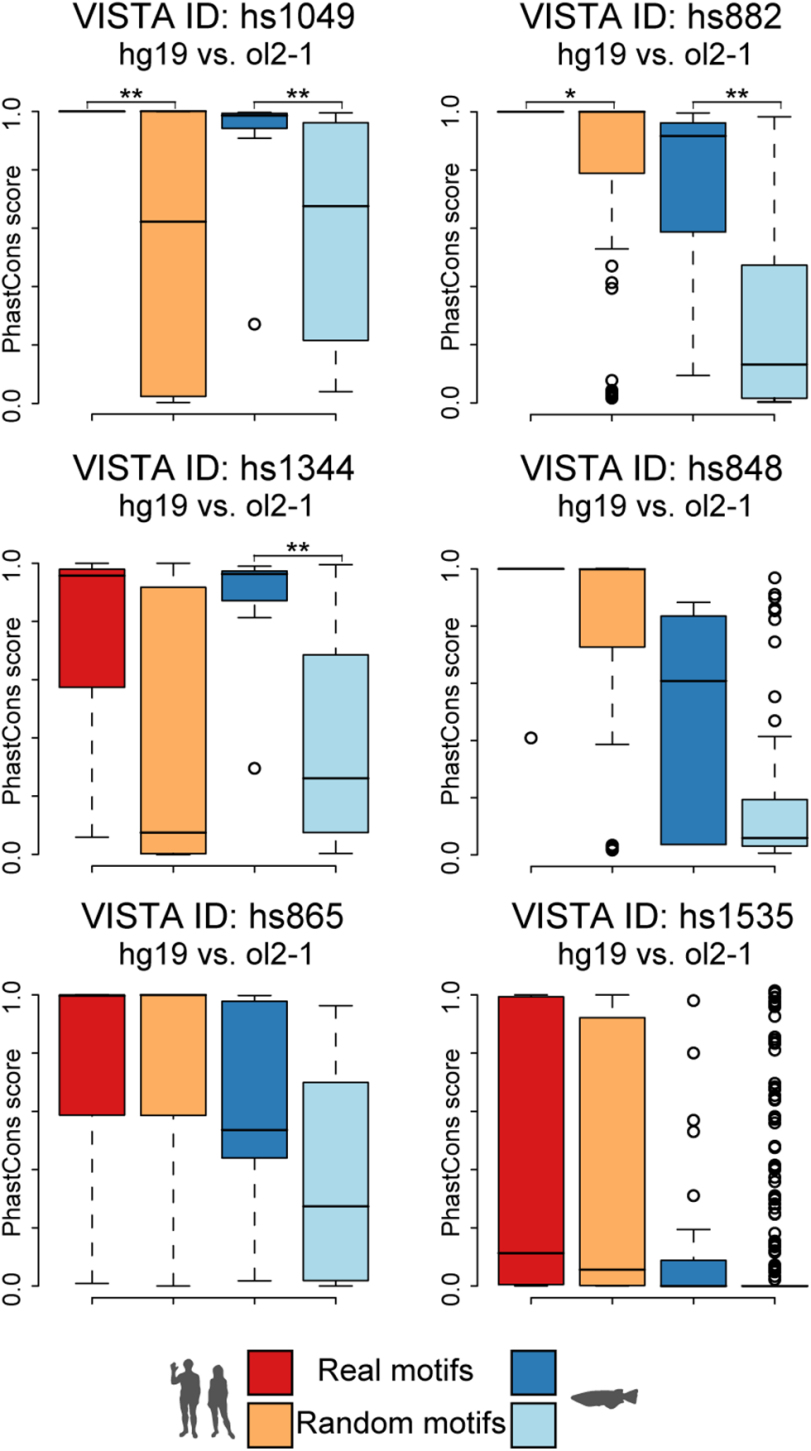
Predicted candidates do not only differ in their motif configuration but also their conservation levels. Hs1344 shows significant motif specific conservation among teleosts, hs1049 and hs882 also among placental mammals. Together with the co-linear configuration and the orthologous gene(s) in flanking position this indicates that the motifs shared between human and medaka are likely to be orthologous counterparts. The random motifs (light colours) show very diverse conservation levels, which are generally lower within both clades.

We therefore subsequently analysed the motif profiles (Fig 2 and S5B Fig) of active human-medaka pairs, seeking clues for a functional importance of the motifs involved in their prediction. We furthermore wanted to find out why these medaka regions are detectable by our method while being missed by the alignment algorithms used. This analysis revealed that almost all active candidates show mainly co-linear motif arrangements in human and medaka with additional rearranged motifs supporting the core signal. Interestingly, some of the motifs provide enough sequence identity and length to result in direct alignment hits between the two species. However, neither of these hits reaches a score level considered significant (i.e. a BlastN bit score above 50) nor is strong enough to rank the corresponding region among the 25 highest in BlastN. Based on the found motif patterns and given the degree of sequence similarity displayed by the motifs involved, we hypothesize that the identified medaka regions evolved mainly by sequence turnover in the spacers regions resulting in a series of conserved–and putatively still functional—motifs. We therefore tested whether the motifs were not only kept conserved between the two species compared but indeed also under specific constraint within their corresponding clades. For this we used clade-specific PhastCons scores [46] for placental mammals and teleosts (http://hgdownload.cse.ucsc.edu/goldenPath/hg19/phastCons46way/placentalMammals/, http://hgdownload.cse.ucsc.edu/goldenPath/oryLat2/phastCons5way/), and calculated the average conservation of the human and medaka motifs. We then compared these scores against randomly selected motif sets extracted from the same genomic region in human or medaka, respectively. This revealed significant differences in conservation for co-linear NASCAR motifs in two human and three medaka regions (p < 0.05, Wilcoxon rank sum test) (Fig 3, Table 2). As expected, none of the rearranged motifs shows such specific conservation when compared to randomly selected sets.

**Table 2:** P-values for Wilcoxon rank-sum-test of motif conservation in placental mammals and teleosts.

Enhancer-candidate pairs (hg19 vs ol2-1)	p-value (Wilcoxon rank-sum test)	
	Placental mammals	Teleost
**hs1049**	0.006**	0.007**
**hs882**	0.021*	0.004**
**hs1344**	0.072	0.002**
**hs848**	0.136	0.071
**hs865**	0.815	0.147
**hs1535**	0.343	0.512

One asterisk (*) denotes p-value ≤ 0.05

two asterisks (**) means p-value ≤ 0.01.

Taken together, these results suggest that each of the enhancer-candidate pairs containing co-linear motif arrangements derived from a common ancestor predating the tetrapod-teleost split. This is further supported by the fact that the genes directly flanking these pairs in human and medaka are orthologous between both species. Different rates of sequence turnover within the enhancer subsequently led to a degree of fragmentation that rendered the medaka CRE invisible for alignment algorithms, thereby hiding their evolutionary relationship. In contrast, score aggregation over constraint and permuted motifs allowed NASCAR to predict these likely functionally equivalent regions despite the observed fragmentation.

### Methods based on TFBS-profiles cannot identify the enhancers found by NASCAR

The previous result indicates that the motifs that NASCAR uses to pinpoint enhancer candidates are under selective pressure and this could be because these motifs are functional transcription factors binding sites. We then wanted to check if methods based on TFBS-profiles could identify these regions as well. For this comparison we chose EEL (Enhancer Element Locator) [24,47] because the software is still maintained and available and it is suitable for search of candidates in vertebrate-sized genomes. For this analysis we use the collection of TFBS motifs from the CORE Vertebrata database Jaspar version 5 [48].

**Fig 4:**
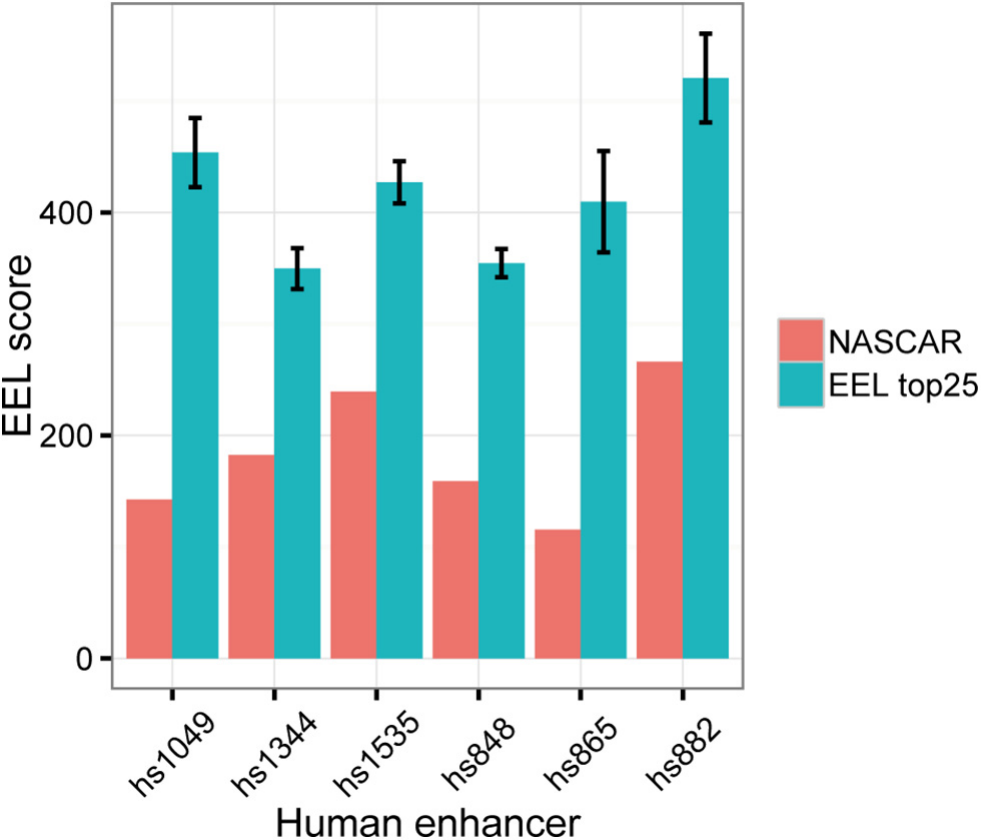
TFBS motif profile search (EEL) misses the enhancers found by NASCAR. Bar plot showing the score assigned by EEL to the enhancer identified by NASCAR and the top 25 ranked regions genome wide with respect to each human enhancer. Error bars show standard deviation.

We used EEL to scan the 24 chromosomes of the medaka genome and took the 25 regions top scored for each human enhancer as query. In no case is the enhancer identified by NASCAR in this top list. More than that, the score assigned by EEL to the validated enhancers is significantly lower than the average score of the top 25 ranked regions by EEL (Fig 4).

However, the medaka enhancers validated here were selected from the list of prediction based on its position near to an orthologous gene. We checked then if the predictions produced by EEL also contain regions with this property. The result is that for none of the human enhancers the 25 predictions are located near human-medaka orthologous genes. This further shows that a TFBS-profile technique cannot perform at a level comparable to NASCAR.

### The identified motifs are functionally relevant for enhancer activity

**Fig 5:**
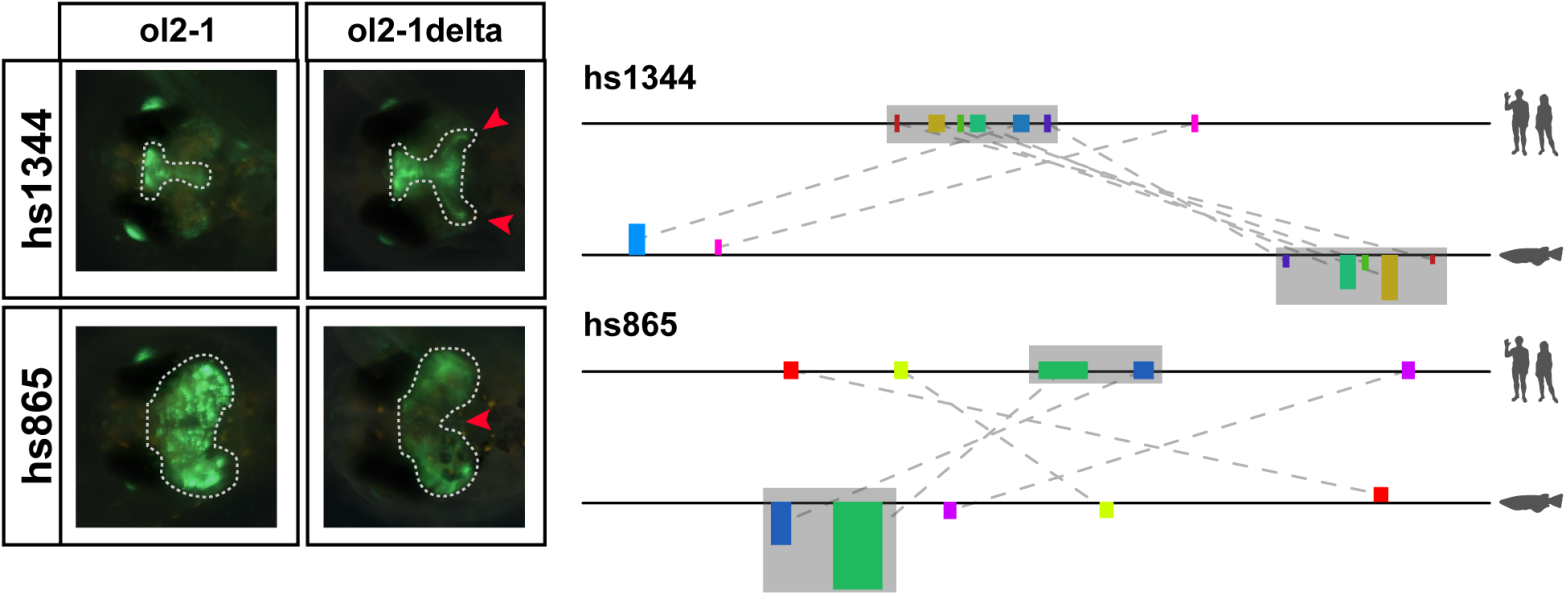
Deletion of conserved motifs (grey area) from the predicted fish regions results in change of enhancer activity in both tested constructs. **Schematic on the right shows the motif configuration in the human and medaka locus for hs1344 and hs865, respectively. The full grey area is deleted from the medaka enhancer and the remaining sequence tested for reporter expression. Images on the left show the reporter activity of the medaka constructs prior to and after the deletion.** Hs1344 ol2-1delta gains two symmetrical domains in the midbrain (red arrowheads), while hs865 ol2-1delta shows a loss of expression in the central part of the original domain.

Conservation of motifs as well as sequence constraint are by themselves neither a prerequisite for function (i.e. regions can diverge without losing function) nor do they necessarily indicate function (i.e. regions might have lost their function although they have been kept largely conserved) [49]. We therefore created deletion constructs lacking some of the identified motifs and tested the effect on the displayed reporter activity. For this we selected two active medaka regions containing motifs only identifiable by NASCAR and removed their conserved co-linear cores (constructs hs1344:ol2-1delta, hs865:ol2-1delta). We subsequently injected these constructs into medaka one cell-stage eggs and analysed the resulting reporter gene activity during development. For each of the constructs two scenarios were possible: if the deleted block acts as an activator we would expect to lose part of the expression pattern, but if the deleted block contains a repressive function its removal should lead to a gain of an expression domain. Interestingly, we observe both scenarios (Fig 5). The first construct, hs1344:ol2-1delta, gained an additional domain (highlighted with the two red arrowheads), indicating that the deleted block represses activity in that domain. On the other hand, hs865:ol2-1delta shows a reduced or absent expression in the central part of the optic tectum, pointing to an activator function of the deleted block. These results hence provide further evidence that the motifs identified by our approach are likely of functional relevance for the corresponding enhancer.

### Additional candidates with reshuffled motif configuration show reporter activity complementary to the initial enhancer

**Fig 6:**
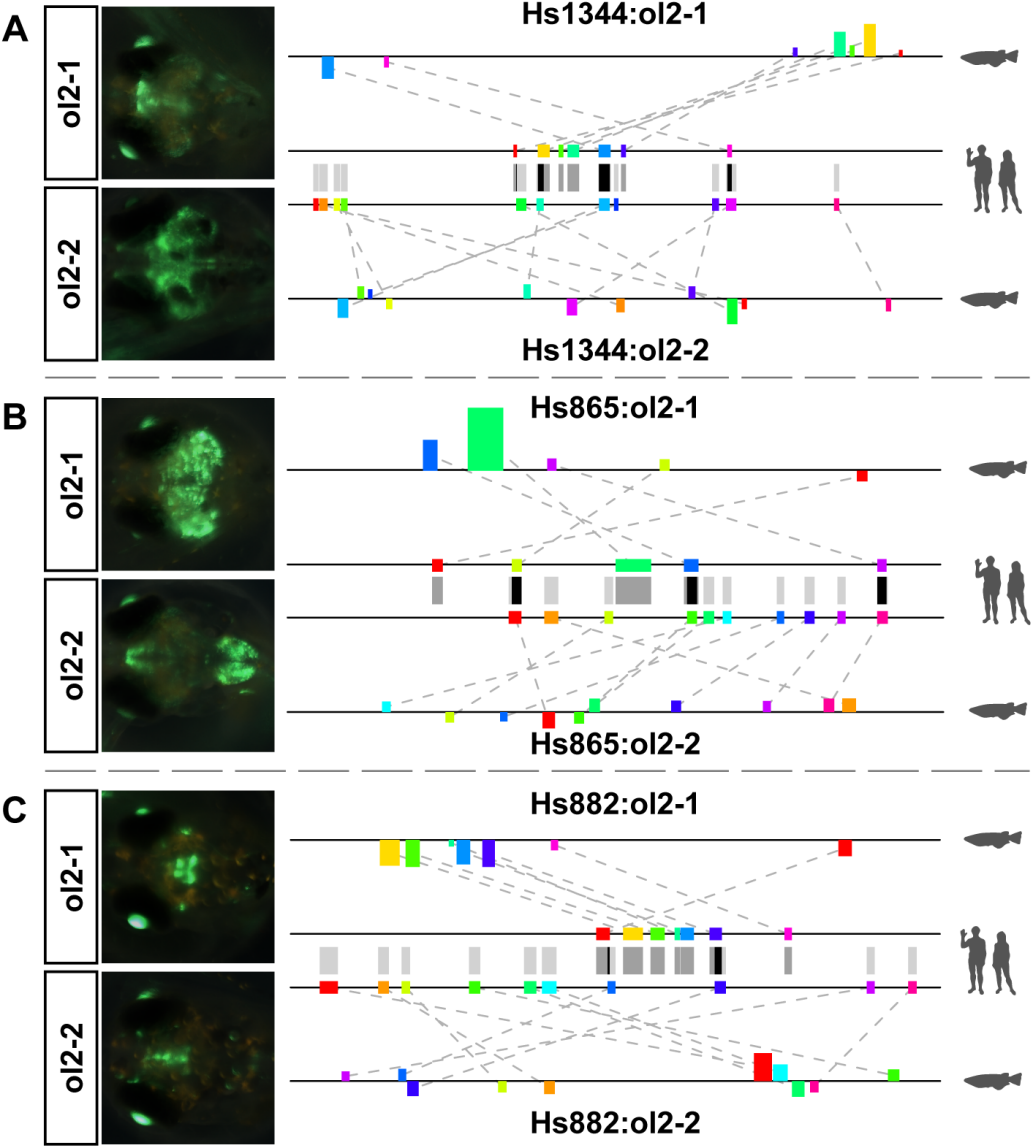
Secondary constructs also show enhancer activity. For each enhancer, the two motif tracks in the middle show the motifs in the human sequence used for prediction of either of the two medaka regions. Grey bars between the tracks are motif projections to the other sequence, black bars indicate motifs shared between both predictions. (A) The secondary construct for hs1344 shows additional expression in more posterior regions of the brain but partially overlaps the domains of the primary construct. (B) The secondary construct for hs865 does not show significant overlap with the primary construct but the combined expression domain of both resembles much better the reported expression domain in mouse (see S5 Fig). (C) For hs882, one additional candidate was found in close proximity to the initial prediction but sharing almost no motifs. Nonetheless, hs882:ol2-2 shows enhancer activity in the brain like hs882:ol2-1.

Most of the predicted enhancers tested so far utilize a co-linear motif configuration and hence provide little support for our ability to predict putative CREs based on rearranged motif patterns. We therefore searched the top 25 candidates per active enhancer pair for regions that show highest possible motif overlap in the human sequence with those of the initial prediction, this time with a reshuffled motif arrangement in medaka. We identified two additional regions for the enhancers hs1344 and hs865 (hs1344:ol2-2, hs865:ol2-2) that not only contain some of the original motifs in a reshuffled configuration, but are also located far away from any gene orthologous to those in the human locus. Hence, the selected regions are neither related by motif configuration nor by common ancestry but only by the used prediction method and the motifs contained. Nonetheless, both regions show strong activity in neuronal areas in the reporter assay (Fig 6A and 6B) like the initially tested candidates for these enhancers. Interestingly, in the case of hs865, the composite expression domain of both medaka enhancers overlaps the domain of the human sequence assayed in mouse, indicating a complementary function of both enhancers.

Besides the additional candidates selected for hs1344 and hs865 in the second round, we also found a second region for one of the other enhancers, hs882, in our initial candidate selection phase. Hs882:ol2-2 is just 4kb away from hs882:ol2-1 and hence shares the same associated flanking gene. However, as its score is lower than that of hs822:ol2-1 it was not tested in the first round. Furthermore, apart from the fact that they share the same gene environment, both predictions are very different. Not only do they have almost none of their motifs in common but they also have very different configurations: while the motifs for hs882:ol2-1 are almost strictly co-linear, hs882:ol2-2 uses a highly reshuffled arrangement. But despite those differences, both predicted regions show strong reporter activity in the midbrain (Fig 6C). While looking for an explanation of why two very different regions sharing almost no motif similarity drive reporter gene expression in the same anatomical structure, we found that hs882:ol2-2 is located in a region matching another element in the VISTA enhancer browser (hs431) that was not included in our test set for two reasons: the human sequence for this human-medaka conserved region showed no enhancer activity in the initial mouse screen [39] and it still aligns strongly to the medaka region containing hs882:ol2-2. By contrast, hs882:ol2-1 seems to have mutated to an extent that left no significant similarity to hs882. It is interesting that two enhancers located that close to each other and driving overlapping reporter activity seem to be under such different levels of evolutionary constraint in the teleosts while both are under strong constraint from human to chicken. It is further remarkable that almost none of the motifs conserved between hs882 and hs882:ol2-1 are also used in hs882:ol2-2. Hence, hs882:ol2-2 might use a very different logic to generate a very similar outcome. Nonetheless, both medaka regions are not only found by NASCAR but also are included in the 25 highest-scoring predictions (rank 1 for hs882:ol2-1 and 12 for hs882:ol2-2, respectively), despite the fact that two very distinct subsets of sequence from just one human enhancer were used.

## Discussion

Since the discovery of the first gene regulatory mechanisms it has been repeatedly suggested that changes in the regulatory landscape, and not genes, might have played–and still play—an important role in phenotypic diversification and perhaps even speciation [9–15]. Unfortunately, our understanding of how specific changes in the DNA sequence impinge on gene regulation is still rather limited. So far, two main diametrically opposed structural models of enhancers, the enhanceosome and the billboard model, exist, both backed up by thorough analysis and validation [1,16–18]. But while the number of known enhanceosomes will likely increase in the coming years (e.g. due to decreasing costs for high throughput sequencing methods and hence a higher number of fully sequenced, high quality genomes available for multiple sequence alignments), the gain of knowledge about billboard enhancers is uncertain. Unfortunately, this class of enhancers is by far more interesting for the investigation of regulatory as well as morphological evolution. While enhanceosomes are likely to be identifiable in many species due to their very rigid structure and the little amount of mutations tolerated, they are at the same time unlikely to contribute largely to morphological diversification. Billboard enhancers on the other hand are described as very flexible and only slightly conserved in their sequence and configuration [17] and are therefore more promising to contribute to changes between species. But this inherent flexibility also makes them more difficult to detect by classical means of sequence comparison like pair-wise or multiple alignments.

We hence looked for a new method of sequence comparison capable of detecting all types of CREs no matter whether they evolve by mutation and/or permutation and at sequence conservation levels too low for classical local alignment algorithms. The principle we present and apply in this study can be seen as one step towards a possible solution as it integrates alignment and alignment-free techniques. Unlike other previously published methods, our concept does not depend on any information other than a given enhancer sequence and the target genome of interest for prediction of putative enhancer regions. It therefore can be applied for all kinds of analyses, from genome-wide scans to local searches. NASCAR can be used either as a pre-screening tool for subsequent more detailed procedures like position weight matrix (PWM) searches or conservation analysis within the target clade, or applied in cases in which no knowledge about the logic of a given enhancer exists. Even if this knowledge is available, NASCAR provides a less biased approach for enhancer prediction, capable of finding additional regions that do not fully fulfil the requirements of an *a priori* defined regulatory logic and would be ignored otherwise. The fact that we are able to not only detect enhancer regions missed by alignment algorithms despite the presence of co-linear motif arrangements, but also find additional regions utilizing partially strongly reshuffled motif configurations, clearly shows the usefulness of our approach. The regions found outside of any orthologous context perhaps demonstrate the latter ability best. These regions show strong activity in neuronal structures in the same way as the putative functionally equivalent candidates, clearly indicating that NASCAR is equally able to predict regulatory regions based on motif profiles that do not derive from residual sequence conservation.

Among all successfully validated predictions, hs1344:ol2-1 might be the best example how each of the implemented principles contributes to the prediction process. This region, directly flanked on both sides by genes orthologous to the flanking genes in human, has a strong and a weak rearranged motif supporting a clearly co-linear motif core displaying very significant motif-specific conservation among the teleosts (p < 0.01, Wilcoxon rank sum test). Among placental mammals however the conservation is not motif-specific (p = 0.07, Wilcoxon rank sum test). Instead, almost the full central region of the enhancer, including motifs and spacer regions, is conserved. This indicates that a relaxation of the selective pressure in the teleosts allowed the spacer regions to mutate, ultimately splitting the enhancer in a cluster of conserved segments, each on its own too small to be picked up even as an insignificant alignment hit (BlastN bit score <50). Similarly, the combined score of all motifs together is too low to position the regions high enough among the NASCAR predictions. However, with support by the implemented pattern detection technique recognizing the co-linear configuration, this enhancer can be ranked high enough to be detected. Hence, all the implemented principles together are necessary to detect this likely functionally equivalent enhancer that is undetectable by standard alignment algorithms.

The fact that many of our likely functionally equivalent predictions contain conserved co-linear configurations can be explained by the chosen input set. For this proof of principle study we needed a collection of validated enhancers that was likely to still exist in medaka, yet diverged enough to contain candidates not predictable by alignment algorithms. We therefore chose the VISTA enhancer set, which mainly contains enhancers under strong evolutionary constraint. Such regulatory elements are usually considered to be of crucial importance [2] and hence should also be present in medaka. However, enhancers that are alignable across mammals and most vertebrates, including birds and amphibians, are inherently structurally rigid and by that unlikely to have rearranged in medaka. Furthermore, we selected predictions in close proximity to orthologous genes to prioritize likely active regions for this proof of principle study. As this criterion derived from the analysis of aligning enhancers we might have indirectly chosen regions that are less likely to rearrange. Nonetheless, the majority of enhancers with a co-linear core (3/5) shows at least one reshuffled motif with a higher score than one or more of the co-linear ones. In addition, 4 of 9 (~44%) successfully validated predictions actually use rearranged motif configurations–like the vast majority of predictions scoring among the top 25 per enhancer. The observation that most rearranged configurations also occur in regions of the medaka genome far from any ortholog to a gene in the human locus is by itself no evidence for a false positive prediction. This is shown not only by two successfully validated rearranged candidates found in regions lacking any obvious ancestral relationship, but also by some very strong alignments in remote locations that were found for enhancers in the aligning set. One of these remote enhancer candidates (hs208) aligns to a locus on chromosome 4 (chr4:4794327–4794857) in medaka with a BlastN bit score of ~700. Strangely, no ortholog (or at least paralog) for a gene in the vicinity of the human enhancer can be found on that chromosome. In contrast, clusters of orthologs are found on chromosomes 20, 21 and 22. While future improvements of the medaka genome and gene annotation might be able to provide an explanation for these findings, it shows that candidate selection by orthologous genes in flanking positions is likely to underestimate the amount of active candidates in our predictions.

Our analysis of two deletion candidates shows that the motifs extracted by NASCAR are indeed of functional relevance for the predicted enhancers. Some of these motifs are also under strong selective pressure that kept them almost identical throughout the ~450Myr of independent evolution between human and medaka. This motif specific constraint however is overshadowed by a high rate of sequence turnover in the surrounding regions that makes the enhancers non-alignable and seemingly non-conserved. Given the results presented here it might be necessary to reconsider the criteria used to define whether or not an enhancer is conserved. Five predicted regions with co-linear motif profiles showed activity in our assay, as well as four with reshuffled arrangements. Furthermore, six constructs drive expression in overlapping tissues compared to the human counterpart, three of them even result in similar patterns. This is in the range of the 30% reported for putative orthologous enhancers conserved between human and zebrafish [50] and clearly indicates that partially co-linear and/or even fully rearranged enhancers might still fulfil the same vital role for organisms in one clade as the fully conserved regions for the species in another. Hence, the current definition of enhancer conservation is more a reflection of the detection methods available than of the actual function of the enhancer itself. Future extensions of the principles presented here will hopefully contribute to the development of a new definition and broaden our understanding of enhancer function.

## Methods

### Fish maintenance

Medaka (Oryzias latipes) stocks were maintained as previously described [51]. In this study only the medaka wild-type Cab line was used. Stock animals were kept in accordance with the German national guidelines (Tierschutzgesetz). Only embryos were treated and always prior to hatching implying that no animal experiments were performed.

### VISTA Enhancer sets

We used the VISTA Enhancer browser [39] (state 2010-12-07) to generate a set of validated human enhancers. For this, we applied its internal search routine to extract all human regions (hg19) that show enhancer activity at stage E11.5 in mouse. If enhancers selected this way are closer than 250nt (distance between enhancer boundaries) to one another, these were considered to be “overlapping” and all of them were discarded. The reasoning behind that decision is that the cloning procedure of the enhancer assay used requires ~300nt flanking sequence up- and downstream of each element which would lead to mixed elements of different enhancers that could confound our results. On the other hand, selecting the union of the overlapping regions would result in sequences too long to be cloned. Following these selection and filtering steps we obtained a final set of 629 fully independent human enhancers.

### Sequence alignments

We retrieved the repeat-masked sequences of all human enhancers as well as the full medaka genome (ol2) from Ensembl using the Ensembl API (v63) and subsequently aligned them using either LastZ [43] (command-line parameters:—noytrim,—inner = 2000,—masking = 40,—chain,—hspthresh = 2200,—ydrop = 3400,—gappedthresh = 6000) or BlastN (NCBI-Blast+ Suite v2.2.25) [44] (ftp://ftp.ncbi.nlm.nih.gov/blast/executables/blast+/2.2.25/) (BlastN command-line parameters: -reward 2, -penalty -3, -gapopen 5, -gapextend 2, -word_size 7, -dust "20 64 1", -soft_masking TRUE). As BlastN, in contrast to LastZ, only reports the highest scoring alignment hit, we used different E-value filters to produce more extensive hit lists for BlastN. These were subsequently filtered to form sets containing either only significantly aligning hits (bit score > = 50) or 25 hits with the highest bit score per enhancer. Ties amongst these 25 were resolved by sorting hits first by the length of the hit in decreasing order and second by chromosomal coordinates in ascending order (alphanumeric sorting for chromosomes).

### Orthologous gene sets

To perform the assignment of orthologous genes to enhancer-candidate pairs we first selected all protein coding genes in the human and medaka genome according to the Ensembl gene build (v63). Based on their transcription start site (TSS), we then assigned all the genes in a window of 1.5Mb up- and down-stream of its boundaries to a given human enhancer. Each of the gene sets generated in this way had to contain at least five genes on each side of its assigned enhancer. In cases where this criterion was not met, we extended the margin on either side to reach the minimum number of genes. Using Ensembl Compara (v63) we then checked for each gene in a given human gene set whether it is orthologous to a gene in the gene set generated for the predicted corresponding medaka region. Paralogs were not considered. Each predicted medaka region was then categorized based on the existence of and the relative position to an orthologous gene. We called a candidate “double flanked” if orthologs of both flanking genes in human also flank the medaka predicted regions, “single flanked” (just one of the flanking genes still in flanking position), “near flank” (an ortholog of a flanking gene in human is still within the set distance and gene cuttoffs in medaka but not directly flanking the prediction), “not flanked” (orthologous gene within the cutoff but not flanking in human), and “not orthologous” (not a single orthologous gene near).

### Motif filtering

To reduce the amount of “noise” signal generated by small and abundant motifs, we determined the threshold for arbitrary matches by scanning a set of variably sized enhancers against the target genome and assessed the relative occurrence of perfect-matching words for all extracted windows. Our analysis shows that words shorter than 12nt not only occur in more than 50% of all windows but also accumulate per window with increasing enhancer size (S2 Fig). Motifs with a score below that of a 12-mer were hence excluded for similarity assessment.

### Profile generation

NASCAR generates motif profiles by first dissecting query (i.e. enhancer) and target sequence into words of variable size that match perfectly between the two sequences. Each word then serves as “seed” for subsequent mismatch extension. Extension is performed independently in both directions (upstream/downstream) using a simple additive match-mismatch scoring function until the motif score drops below 0 (i.e. accumulated mismatches score higher than all matching nucleotides). Both extensions are then truncated to the shortest, highest scoring region starting at the seed and merged to form the final motif. Each motif is thereby regarded as a mismatch-containing word similar to perfect matching words in alignment-free algorithms. The same procedure is repeated for the reverse complement target sequence and both generated profiles are merged. This profile is then filtered to remove motifs overlapping in either the query or the target sequence. Starting with the target sequence, motifs are first ranked by their score and then mapped to the target, starting with the highest scoring. The score of each motif is determined by

$$m_{i}^{Score}=(p_{i}\cdot s_{p}+q_{i}\cdot s_{q})\cdot m_{i}^{Length}$$ (1)

with M = {*m*_*1*_, *m*_*2*_, …, *m*_*w*_}, M = all motifs in target window, *p*_*i*_, *q*_*i*_ = matching/mismatching nucleotides in *m*_*i*_; *s*_*p*_, *s*_*q*_ = scores for matching/mismatching nucleotides. Overlapping motifs are truncated to their next matching nucleotide and rescored as long as they still contain a perfect matching region of at least minimum seed size (*k* = 8nt by default). The only exceptions are motifs of equal score, which are allowed to overlap. This filtered motif set forms the final target profile.

### Score calculation

The NASCAR score is calculated for every window sliding along the given target sequence as done for alignment-free algorithms (default: window size = enhancer length, stepping 25%). For this, all motifs in the target profile that are either contained in the window area or overlap its boundaries are selected. The extracted profile is then filtered for word overlap in the query sequence identical to the target filtering procedure, resulting in the fully filtered profile. All words above or equals the minimal score cutoff (default: score of a perfect matching 12-mer) are then used to calculate the final similarity score for the target window (“*Score*_*PURE*_”, *M*_*Valid*_ = all motifs above threshold after filtering):

$$Score_{PURE}=\sum_{m_{i}\in M_{Valid}}m_{i}^{Score}$$ (2)

### Pattern detection

In a parallel approach, clusters of co-linear motifs within a certain distance and query-target shift (default: *Max*_*Distance*_ = 200nt, *Max*_*Shift*_ = 25nt) are traced in the fully filtered profile. For this, all motifs above the score threshold are ranked again in decreasing order. Then, starting at the strongest motif, two elliptical motif-spaces along the current motif diagonal and overlapping in one focus are computed, with the motif placed in the overlapping focus (S3 Fig). The distance between the two foci in query or target corresponds thereby to the enhancer length. This allows detection of patterns spanning the full window size. All motifs located with their centre within these motif spaces and an inter-motif distance (i.e. distance between end of one motif and start of the subsequent one) smaller or equals *Max*_*Distance*_ are combined into a motif pattern. Once a motif is assigned to a pattern it is removed from the motif list and cannot be used for any other pattern. All patterns of three or more motifs containing at least two words scoring at or above the threshold level are valid. This allows inclusion of motifs even below threshold as long as the previous requirement is met. For each motif pattern consisting of m motifs the mean motif distance and the pattern shift (i.e. the weighted midline of the pattern) are calculated:

$$M_{p}=\left\{m_{1},m_{2},\cdots ,m_{n}\right\}$$ (3)

$$Mean_{Distance}=\frac{\sum_{i=1}^{\left|M_{p}\right|-1}\left(m_{i+1}^{Start}-m_{i}^{End}\right)}{\left|M_{p}\right|-1}$$ (4)

$$Pattern_{Shift}=\frac{\sum_{m_{i}\in M_{p}}\left(m_{i}^{Length}\cdot m_{i}^{Shift}\right)}{\sum_{m_{i}\in M_{p}}m_{i}^{Length}}$$ (5)

Then, two correction factors are calculated and used to assign a weight to each motif in the pattern:

$$Factor_{Distance}=1-\frac{Mean_{Distance}}{Max_{Distance}}$$ (6)

$$Factor_{Shift}^{i}=1-\frac{\left|m_{i}^{Shift}-Pattern_{Shift}\right|}{2\cdot Max_{Shift}}$$ (7)

$$m_{i}^{Weight}=mean(Factor_{Distance},Factor_{Shift}^{i})$$ (8)

The final pattern score, i.e. the weighted sum of all contained motifs, is then added to the previously computed “*Score*_*PURE*_” to form the final score (“*Score*_*COMB*_”):

$$Pattern_{Score}=\sum_{i=1}^{\left|M_{p}\right|}m_{i}^{Score}\cdot m_{i}^{Weight}$$ (9)

$$Score_{COMB}=Score_{PURE}+Pattern_{Score}$$ (10)

### Definition of predicted regions

All regions that have a NASCAR score above a set threshold were considered as predictions. These regions are continuous intervals in the target sequence starting at the first window scoring above the threshold and ending as soon as the score drops below this limit. Default threshold is three times the median absolute deviation (MAD) above the median NASCAR score for all windows assessed for the corresponding enhancer. Regions were called individually for the basic (“PURE”) score and the score including patterns (“COMB”).

### Random motif sets

We generated random motif sets by randomly extracting sequence segments of the same size as the real motifs from the given enhancer/candidate region in either human or medaka. Individual segments were thereby not allowed to overlap. This way, 10 independent random sets for each of the validated enhancer-candidate pairs were generated.

### Evolutionary conservation

We analysed the conservation of NASCAR motifs by averaging across the compiled conservation information of all nucleotides forming a motif. The conservation data (PhastCons scores) for placental mammals (mammal sub set of the 46-way MultiZ vertebrate alignment) and teleosts (5-way MultiZ alignment) was obtained from the UCSC Genome Browser (http://hgdownload.cse.ucsc.edu/goldenPath/hg19/phastCons46way/placentalMammals/, http://hgdownload.cse.ucsc.edu/goldenPath/oryLat2/phastCons5way/).

### Prediction of enhancers with EEL

We downloaded EEL (Enhancer Element Locator) from its GitHub repository (https://github.com/kpalin/EEL) as of 27^th^ of January 2015. As TFBS motifs we used the CORE Vertebrata database from Jaspar version 5. We used EEL in its command line mode with the following command structure:

eel -as <humanSeq> -as <medakaSeq> -am <MotifsFolder> -getTFBS -align -sa -no-gui -savealign <outputFile>

In all cases the input sequences are repeat-masked and obtained as mentioned above. We run EEL independently for each medaka chromosome and included the parameter–*more 10* to allow more predictions per run. Then, for each query human enhancer we sorted all the reported predictions and selected the top 25 with highest score.

### Cloning

We used an Hsp70 basal promoter driving eGFP in a cassette flanked by ISceI restriction sites for efficient integration into the genome at early stages of embryonic development[41]. We amplified all fragments from human or medaka genomic DNA by PCR using Phusion DNA Polymerase and the primers specified in S2 Table. For detection of their putative enhancer activity we ligated the fragments containing putative regulatory elements (or control sequences) upstream of the HSP70 promoter. Resulting constructs were tested by restriction digest and injection grade DNA was prepared following the MidiPrep protocol of the QIAGEN Plasmid Purification kit.

### Microinjection

Injections were performed following the meganuclease approach as described previously [52,53]. The efficiency of the meganuclease mediated transgenesis in medaka results in uniform expression patterns and a low degree of mosaicism already in the injected generation. This facilitates the effective detection of enhancer activity even if active only in few cells. In brief, medaka embryos were microinjected into the cytoplasm at the one cell stage. The concentration of the reporter construct was at 10 ng/μl. DNA was diluted in 1x ISceI buffer, containing ISceI enzyme (NEB) at a concentration of 1U/μl. The DNA/enzyme mix was kept on ice prior to microinjection. For each construct at least 50 surviving embryos were scored.

To score successful injection we monitored baseline activity of the HSP70 promoter in the lens as injection control allowing to discriminate between inactive putative enhancer elements and failed injections. An enhancer was considered to be active if at least 35% (average 64%) of all lens positive fish showed a consistent expression pattern. Images of injected embryos were taken on an OLYMPUS MVX10 binocular at 4x magnification using a LEICA DFC500 camera. See S3 Table for a summary of the injection experiments.

### Deletion constructs

We generated deletion constructs by following the procedure described in [54]. In short, we used additional primers directed away from the deletion site together with the original primers for the constructs. Each of the deletion primers thereby also contains a 13-15nt fragment 5’ of its start position in the construct, which is complementary to the sequence on the other flank of the deletion site. Using always one standard and one deletion primer led to amplification of just the flanking regions in the construct. After purification of the fragments, both were used in one reaction together with the standard primers leading to a fusion construct lacking the targeted deletion site.

### Ethics Statement

All fish are maintained in closed stocks at Heidelberg University. In this study only the medaka (*Oryzias latipes*) wild-type Cab line was used. Stock animals are kept according to local animal welfare standards (Tierschutzgesetz §11, Abs. 1, Nr. 1, husbandry permit number 35–9185.64/BH Wittbrodt) and in accordance with European Union animal welfare guidelines. Only embryos were treated and always prior to hatching implying that no animal experiments were performed. The fish facility is under the supervision of the Interfacultary Biomedical Faculty (IBF) of the University of Heidelberg.

## Supporting Information

S1 Fig Illustration of problems of motif-based sequence comparison. Best case-scenario of an enhancer consisting only of directly adjacent functional motifs (uni-coloured boxes). Motifs are extracted independently from query and target sequence in an overlapping fashion with each motif being shifted by one nucleotide compared to the previous. (A) In case both sequences are identical, the resulting profiles are maximally overlapping as each motif in one sequence has a corresponding match in the other. (B) Single nucleotide changes in one of the two sequences (positions marked by white “x” on black background) however remove all motifs overlapping this event from the matching profile (empty boxes). Each point mutation can thereby delete up to k (k = motif size) motifs (B, upper panel). Scattered mutations across the entire sequence can hence lead to a strong reduction of matching motifs and thereby hide all existing similarity. This is clearly different to alignment algorithms, which remain mostly unaffected as long as at least one continuous matching region (“seed”) exists that allows extension across the contained mutations (B, lower panel). (C) Permutation of individual motifs can have a very similar effect on the matching profile, as all motifs overlapping the boundary will not exist anymore after the position change. The strength of a permutation event thereby depends on the context (C, upper vs. lower panel). The main problem however is that the majority of motifs are context dependent (A to C, mixed coloured motifs). By that, the maximum signal intensity can be only reached if co-linearity is kept which is contrary to the idea of using alignment-free methods for the detection of rearranged regions. (D) Furthermore, the alignment-free principle by itself cannot discriminate between important and unimportant motifs. As a result, an arbitrary sequence can generate as many matching motifs as a permuted one that has kept its functionality (D, upper vs. lower panel). But as more context dependent than functional motifs exist within billboard enhancers, permuted arrangements of non-functional motifs are much more likely to happen.
(TIF)

S2 Fig Determination of “noisy” words. Enhancers of several sizes were used to scan the full medaka genome and determine the size and type of motifs occurring per window depending on the size. (A) Perfect matching motifs smaller or equals 11nt (vertical blue line) occur in more than 50% of all windows in the medaka genome (horizontal red line). (B) Additionally, matching motifs up to that size accumulate per window with increasing window size, indicating that they are likely arbitrary matches between both sequences.
(TIF)

S3 Fig Pattern detection. For each motif above threshold (grey bars) two elliptic spaces (upstream ellipse drawn only dotted; a,b,e are standard values of ellipses) are computed with the motif being located in the overlapping focus point (FC). All motifs within those spaces that are also within the set inter-motif distance form a pattern even if they are below the score threshold (orange bar). At least three motifs have to be combined in that way, two of them above score threshold.
(TIF)

S4 Fig Definition of the gene environment per enhancer/prediction. For each enhancer/prediction (filled triangles) a region 1.5Mb up- and downstream was selected (correspondingly coloured arcs). In case the up- or downstream region contained less than five genes (upper sequence, left side), additional genes in the same direction were included to reach a minimum of five. Genes orthologous between the human and medaka set (black rectangles, flanking genes in purple) are connected with dashed lines (non-orthologous genes in grey) Following the colour gradient from dark blue to light blue, predictions are: “double flanked”, “single flanked”, “near the ortholog of a former flanking gene”, “near an orthologous (formerly not flanking) gene”.
(TIF)

S5 Fig Selected enhancer elements. Eight selected human VISTA enhancers show activity in the medaka reporter assay (column “Human in Medaka” in A), indicating that the “trans” environment is still capable of activating these enhancers despite the ~450Myr of independent evolution. Six of eight predicted medaka regions also show enhancer activity (column “Medaka in Medaka” in A). Activity is visible in most cases in the brain and other neuronal structures. Interestingly, in most of the cases the reported expression pattern of the human enhancer in mouse (column “Human in Mouse” in A) resembles the expression pattern of the human or medaka sequences in medaka. Lens activity is part of the reporter construct and allows to distinguish between successful and negative injections. All pictures are taken at 10 days post injection (10dpi). Mouse pictures were downloaded from http://enhancer.lbl.gov/. (B) Comparison of the known human enhancer sequence and the predicted enhancer in medaka. The coloured boxes represent the motifs identified by NASCAR to assess the similarity of each pair. Upper track always displays the motif positions in the human sequence (colour coded by position), lower track shows the configuration in the medaka region. All Motifs are draw in size relative to the used window size. Motif heights in the lower track represent the motif score, orientation (up/down) indicates the relative orientation (forward/reverse) compared to the query sequence.
(TIF)

S1 Table Alignment hits. BlastN alignment hit for the human enhancer on the medaka genome. DF = “double flanked”, SF = “single flanked”, NF = “no flanked”.
(XLSX)

S2 Table List of cloning primers. (XLSX)

S3 Table Summary of the injection experiments. (XLSX)

S1 File Supplementary methods. (DOCX)
