# Correction: Handling Permutation in Sequence Comparison: Genome-Wide Enhancer Prediction in Vertebrates by a Novel Non-Linear Alignment Scoring Principle

**DOI:** 10.1371/journal.pone.0143989

**Published:** 2015-11-23

**Authors:** Dirk Dolle, Juan L. Mateo, Michael P. Eichenlaub, Rebecca Sinn, Robert Reinhardt, Burkhard Höckendorf, Daigo Inoue, Lazaro Centanin, Laurence Ettwiller, Joachim Wittbrodt

The following information is missing from the Funding section: The authors acknowledge the financial support of the Deutsche Forschungsgemeinschaft and Ruprecht-Karls-Universität Heidelberg within the funding programme Open Access Publishing.
