# Supplementary figures and images for: Handling Permutation in Sequence Comparison: Genome-Wide Enhancer Prediction in Vertebrates by a Novel Non-Linear Alignment Scoring Principle
Source: PLoS One. 2015 Oct 27;10(10):e0141487. doi: 10.1371/journal.pone.0141487 (PMC4624239; doi:10.1371/journal.pone.0141487)

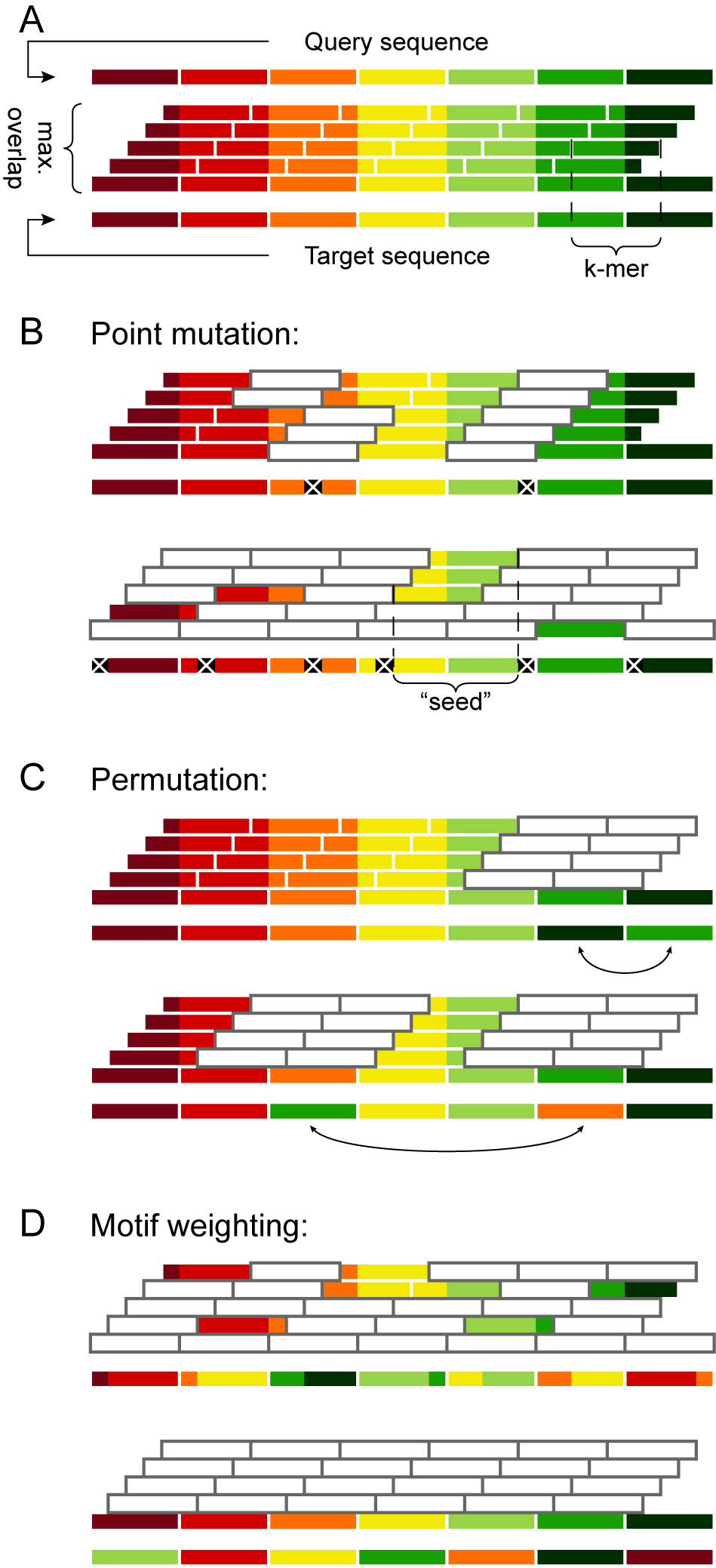

Supplement: S1 Fig — Best case-scenario of an enhancer consisting only of directly adjacent functional motifs (uni-coloured boxes). Motifs are extracted independently from query and target sequence in an overlapping fashion with each motif being shifted by one nucleotide compared to the previous. (A) In case both sequences are identical, the resulting profiles are maximally overlapping as each motif in one sequence has a corresponding match in the other. (B) Single nucleotide changes in one of the two sequences (positions marked by white “x” on black background) however remove all motifs overlapping this event from the matching profile (empty boxes). Each point mutation can thereby delete up to k (k = motif size) motifs (B, upper panel). Scattered mutations across the entire sequence can hence lead to a strong reduction of matching motifs and thereby hide all existing similarity. This is clearly different to alignment algorithms, which remain mostly unaffected as long as at least one continuous matching region (“seed”) exists that allows extension across the contained mutations (B, lower panel). (C) Permutation of individual motifs can have a very similar effect on the matching profile, as all motifs overlapping the boundary will not exist anymore after the position change. The strength of a permutation event thereby depends on the context (C, upper vs. lower panel). The main problem however is that the majority of motifs are context dependent (A to C, mixed coloured motifs). By that, the maximum signal intensity can be only reached if co-linearity is kept which is contrary to the idea of using alignment-free methods for the detection of rearranged regions. (D) Furthermore, the alignment-free principle by itself cannot discriminate between important and unimportant motifs. As a result, an arbitrary sequence can generate as many matching motifs as a permuted one that has kept its functionality (D, upper vs. lower panel). But as more context dependent than functional motifs exist within b [file pone.0141487.s001.tif]

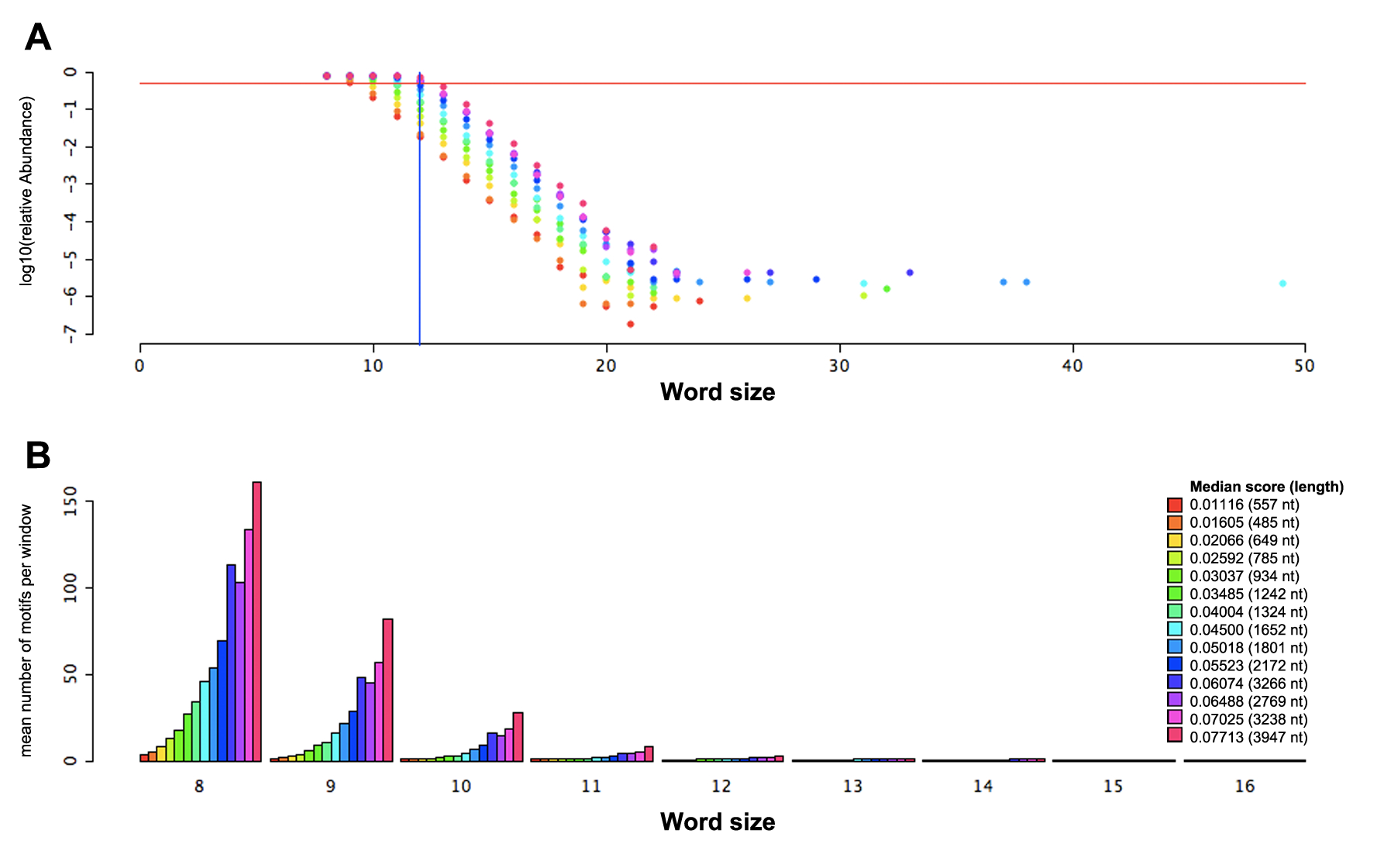

Supplement: S2 Fig — Enhancers of several sizes were used to scan the full medaka genome and determine the size and type of motifs occurring per window depending on the size. (A) Perfect matching motifs smaller or equals 11nt (vertical blue line) occur in more than 50% of all windows in the medaka genome (horizontal red line). (B) Additionally, matching motifs up to that size accumulate per window with increasing window size, indicating that they are likely arbitrary matches between both sequences. (TIF) [file pone.0141487.s002.tif]

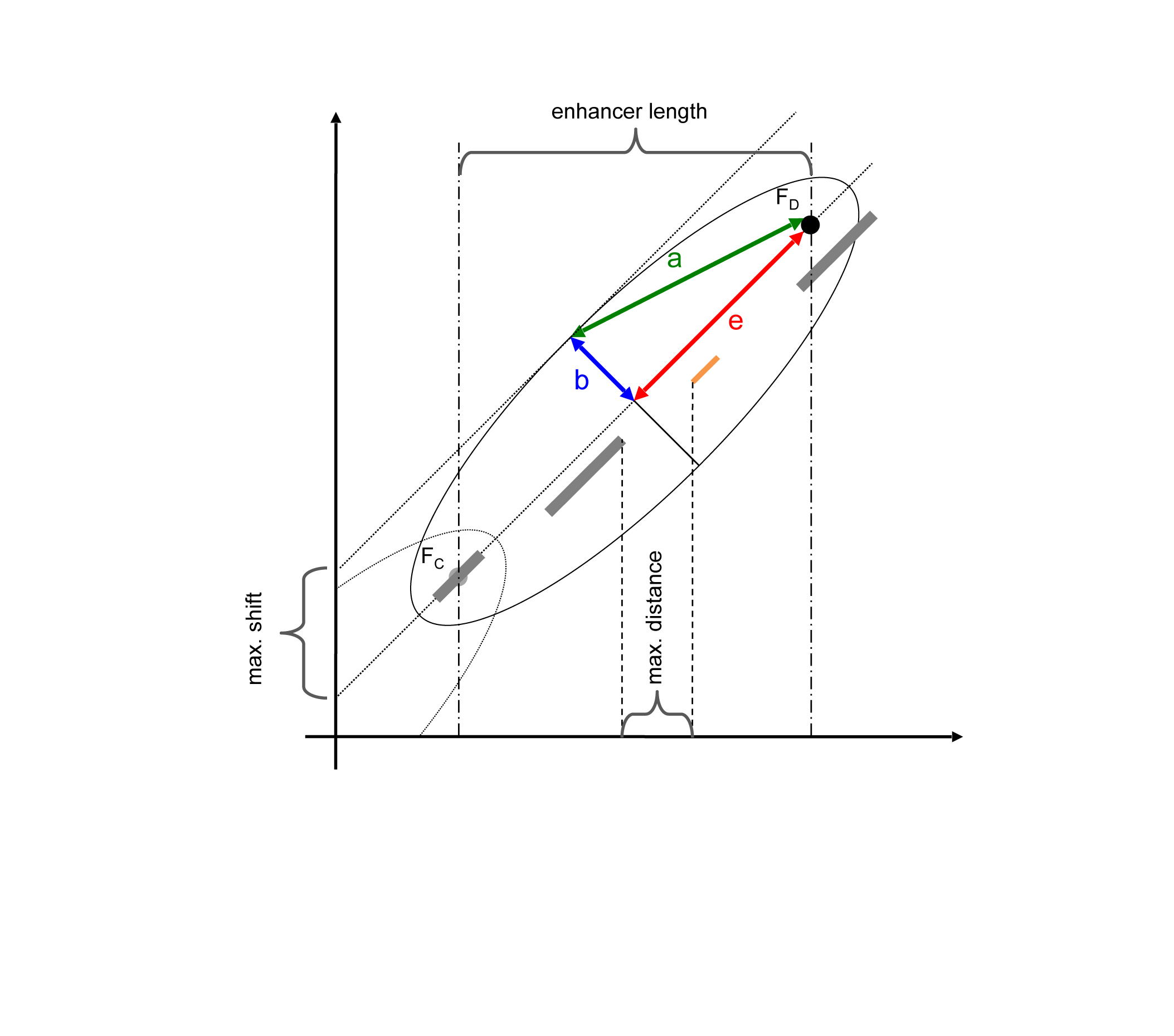

Supplement: S3 Fig — For each motif above threshold (grey bars) two elliptic spaces (upstream ellipse drawn only dotted; a,b,e are standard values of ellipses) are computed with the motif being located in the overlapping focus point (FC). All motifs within those spaces that are also within the set inter-motif distance form a pattern even if they are below the score threshold (orange bar). At least three motifs have to be combined in that way, two of them above score threshold. (TIF) [file pone.0141487.s003.tif]

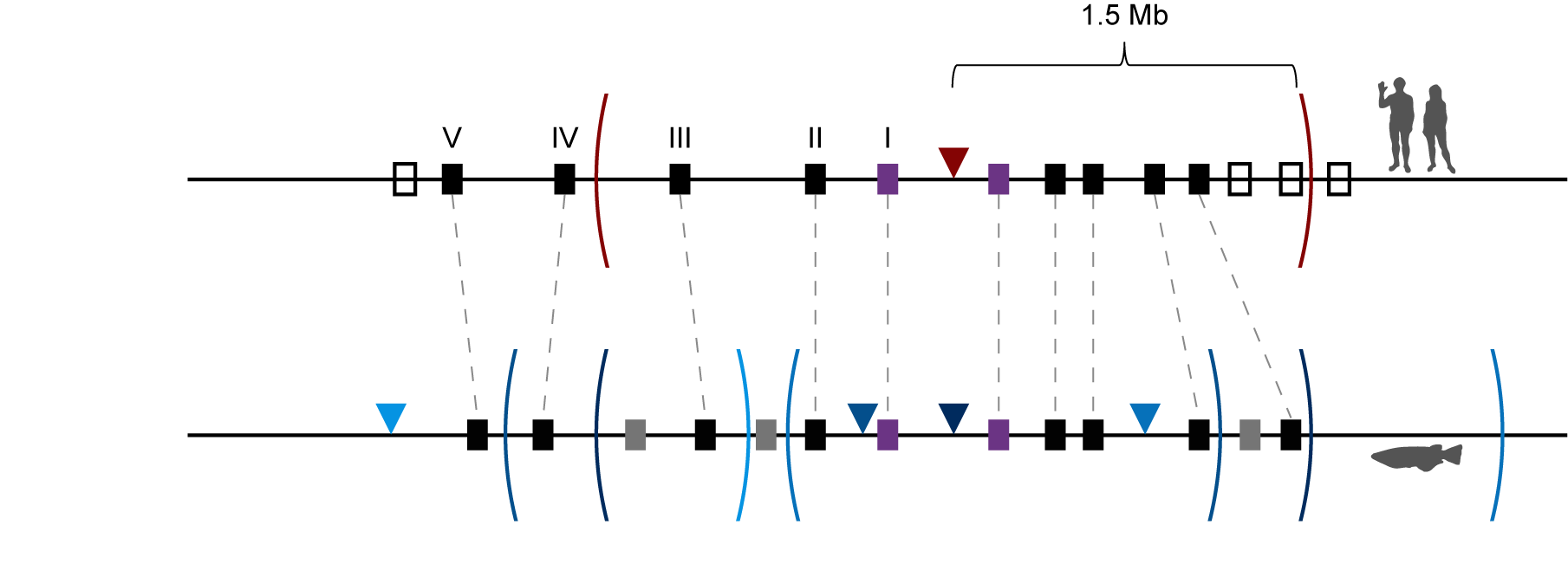

Supplement: S4 Fig — For each enhancer/prediction (filled triangles) a region 1.5Mb up- and downstream was selected (correspondingly coloured arcs). In case the up- or downstream region contained less than five genes (upper sequence, left side), additional genes in the same direction were included to reach a minimum of five. Genes orthologous between the human and medaka set (black rectangles, flanking genes in purple) are connected with dashed lines (non-orthologous genes in grey) Following the colour gradient from dark blue to light blue, predictions are: “double flanked”, “single flanked”, “near the ortholog of a former flanking gene”, “near an orthologous (formerly not flanking) gene”. (TIF) [file pone.0141487.s004.tif]

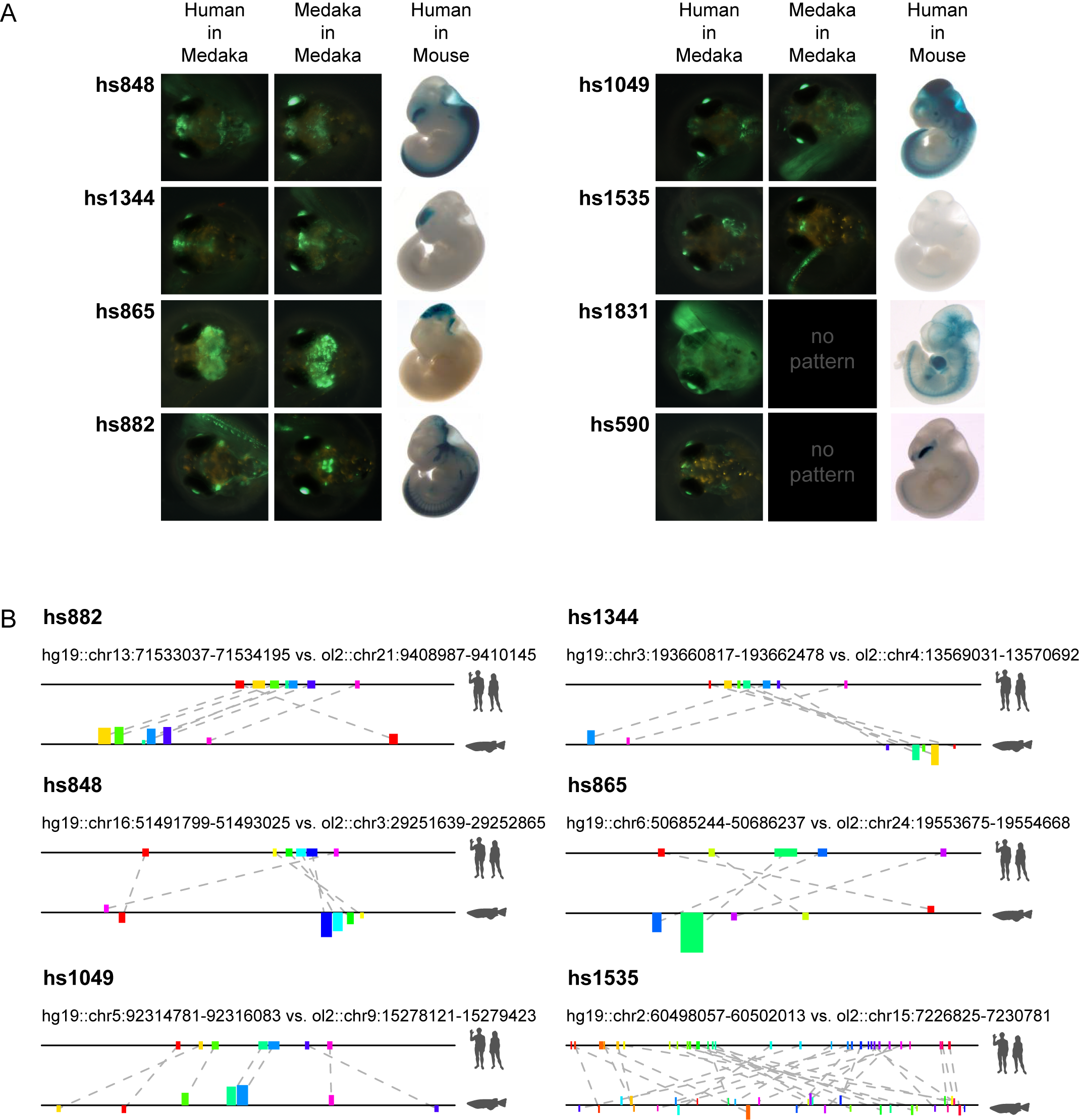

Supplement: S5 Fig — Eight selected human VISTA enhancers show activity in the medaka reporter assay (column “Human in Medaka” in A), indicating that the “trans” environment is still capable of activating these enhancers despite the ~450Myr of independent evolution. Six of eight predicted medaka regions also show enhancer activity (column “Medaka in Medaka” in A). Activity is visible in most cases in the brain and other neuronal structures. Interestingly, in most of the cases the reported expression pattern of the human enhancer in mouse (column “Human in Mouse” in A) resembles the expression pattern of the human or medaka sequences in medaka. Lens activity is part of the reporter construct and allows to distinguish between successful and negative injections. All pictures are taken at 10 days post injection (10dpi). Mouse pictures were downloaded from http://enhancer.lbl.gov/. (B) Comparison of the known human enhancer sequence and the predicted enhancer in medaka. The coloured boxes represent the motifs identified by NASCAR to assess the similarity of each pair. Upper track always displays the motif positions in the human sequence (colour coded by position), lower track shows the configuration in the medaka region. All Motifs are draw in size relative to the used window size. Motif heights in the lower track represent the motif score, orientation (up/down) indicates the relative orientation (forward/reverse) compared to the query sequence. (TIF) [file pone.0141487.s005.tif]
