# Supplementary material for: Handling Permutation in Sequence Comparison: Genome-Wide Enhancer Prediction in Vertebrates by a Novel Non-Linear Alignment Scoring Principle
Source: PLoS One. 2015 Oct 27;10(10):e0141487. doi: 10.1371/journal.pone.0141487 (PMC4624239; doi:10.1371/journal.pone.0141487)
Supplement: S1 File — (DOCX) [file pone.0141487.s009.docx]

**Supplementary methods**

*Analysis of the alignment-free principle*

The application of alignment-free algorithms for the task of enhancer prediction starts with a serious dilemma. A priori, the type, number, and position of the functional motifs are unknown. Hence, k-mers are extracted at every position in a given enhancer reasoning that this way all contained functional motifs will be included in the final profile. Obviously, this assumption greatly simplifies TFBS as perfect matching words of equal length independent of the TF binding to it – which clearly is not the case. The vast majority of TFs can bind to a variety of sequences (i.e. words) which taken together form one to many motifs, each with fixed and other more variable positions. Therefore, in case a given enhancer contains several variations of a TFBS whereas the corresponding element in the target region utilizes multiple instances of just one, the resulting scores can be very different. On top of that, the classical profile generation procedure also makes a second, even more problematic assumption. Extracting k-mers at every possible nucleotide position treats all k-mers as if they are independent of each other. In reality, however, almost every k-mer generated in this way shares k-1 nucleotides with its previous or following neighbour, respectively. This results in three important limitations in the alignment-free principle that severely impact on its ability to assess similarity between two given sequences (Suppl. Figure 1). Firstly, a single nucleotide change in one of the sequences can remove k words from the set of shared k-mers by replacing them with their 1-neighbours (i.e. sequences that differ from a given k-mer by just one nucleotide) (Suppl. Figure 1B). Secondly, even in the optimal case that two functional k-mers are located directly side-by-side, they form k-1 meaningless “bridging” k-mers that will be lost in the case of rearrangement and replaced by twice as many new words without any functional importance (Suppl. Figure 1C). This way, both processes not only make the two profiles less similar (even the bridging k-mers connecting the two functional sites would have contributed to the overall similarity despite the fact that they have no function on their own), they also make them more dissimilar at the same time. Although these two concepts might seem to be identical at first glance, in reality they are not. Each of them is based on a separate subset of the word profile – a fact that is used by several metrics. The observation that bridging k-mers between adjacent functional sites also contribute to the overall profile similarity highlights the third important drawback of the profile generation process: the excess of non-functional k-mers in the final profile (Suppl. Figure 1A). Even enhancers composed only of directly adjacent regulatory TFBS (e.g. enhanceosomes) contain almost k-1 times more non-functional than functional k-mers (assuming that all TFBS are indeed of size k). In reality, especially in billboard enhancers, this ratio is very likely to be worse. Hence, only a small fraction of words in the final profile should actually be used for enhancer prediction. However, without additional information this fraction cannot be identified.
